# Supplementary material for: Glycine Supplementation in Obesity Worsens Glucose Intolerance through Enhanced Liver Gluconeogenesis
Source: Nutrients. 2022 Dec 24;15(1):96. doi: 10.3390/nu15010096 (PMC9823780; doi:10.3390/nu15010096)
Supplement: Supplementary file 1 [file nutrients-15-00096-s001.zip › nutrients-2096517-supplementary.pdf]

Table S1 – Metabolite concentrations measured by NMR in the liver of SD, HFHS and HFHS-GLY-fed mice.

Metabolite concentration (nmol/ 100 mg tissue)	**SD**	**HFHS**	**HFHS-GLY**	ANOVA p-value
Glycine	119.9±4.0	91.6±6.2**	110.6±6.7	P<0.01
sn-Glycero-3-	53.8±5.7	35.4±3.7	40.8±6.4	NS
phosphocholine	8.9±0.6	8.1±0.4	9.1±0.7	NS
Methionine	7.3±0.9	14.9±2.0**	16.9±1.5***	P<0.01
Choline	9.8±0.7	27.5±4.0***	28.4±3.1***	P<0.0001
O-Phosphocholine	0.8±0.1	1.1±0.1	1.5±0.3	NS
Sarcosine	2.1±0.2	2.1±0.3	2.4±0.4	NS
N,N-Dimethylglycine	22.0±3.1	25.2±3.3	23.9±2.5	NS
Betaine	1.7±0.2	1.5±0.2	2.2±0.3	NS
Pyruvate	47.2±4.0	42.9±2.9	52.7±5.3	NS
Succinate	22.6±2.5	22.8±2.3	27.8±2.5	NS
Malate	2.7±0.2	2.4±0.3	2.9±0.3	NS
3-Hydroxyisovalerate	12.1±0.7	13.3±1.3	14.0±0.7	NS
Leucine	6.9±0.4	7.1±0.8	7.5±0.4	NS
Isoleucine	11.4±0.8	13.2±1.3	13.5±0.7	NS
Valine	54.5±5.4	69.2±7.5	70.2±3.0	NS
Glutamate	112.4±9.5	95.1±6.3	97.1±8.6	NS
Alanine	597.3±58.8	453.7±16.4	504.1±51.9	NS
Lactate	41.8±2.9	30.1±2.8*	34.2±2.3	P<0.05
Glycerol	1677±65	1642±115	1813±102	NS
Glucose	2.3±0.1	1.7±0.2*	2.1±0.2	P<0.05
N-Acetylcysteine	4.6±0.2	5.0±0.1	4.4±0.6	NS
Glutathione				

Comparison vs. SD: *p<0.05; **p<0.01; ***p<0.001

Table S2 – Metabolite concentrations measured by NMR in the *gastrocnemius* muscle of SD, HFHS and HFHS-GLY-fed mice.

Metabolite concentration (nmol/ 100 mg tissue)	**SD**	**HFHS**	**HFHS-GLY**	ANOVA p-value
Glycine	139.6±4.3	131.1±7.7	187.9±5.9***,$$$	P<0.0001
Choline	14.7±1.0	13.6±0.3	15.5±0.6	NS
Sarcosine	0.40±0.02	0.30±0.02*	0.35±0.02	P<0.05
Pyruvate	6.5±0.4	5.6±0.4	5.9±0.2	NS
Succinate	25.7±1.9	25.4±3.0	25.6±2.3	NS
3-Hydroxyisovalerate	2.4±0.1	2.5±0.1	2.5±0.1	NS
Leucine	6.4±0.3	6.8±0.5	7.7±0.2*	P<0.05
Isoleucine	5.0±0.3	4.5±0.3	5.2±0.1	NS
Valine	10.7±0.5	10.0±0.4	11.2±0.3	NS
Glutamate	81.4±4.3	60.5±4.4**	68.7±1.5	P<0.01
Alanine	118.2±6.8	140.9±4.0**	108.1±2.7$$$	P<0.001
Lactate	893.6±79.2	1060.7±83.4	740.3±18.6$$	P<0.01
Glycerol	31.9±4.2	38.4±2.7	44.2±3.6	NS
Glucose	41.1±4.1	58.6±10.0	58.4±5.5	NS
Glutathione	15.4±1.0	18.6±1.4	15.1±0.8	NS

Comparison vs. SD: *p<0.05; **p<0.01; ***p<0.001. Comparison vs. HFHS: $$p<0.01; $$$p<0.001
